# Learning Processes as Key for Success in Workplace Health Promotion Interventions in Health Care

**DOI:** 10.3389/fpubh.2020.576693

**Published:** 2020-11-10

**Authors:** Andrea Eriksson, Lotta Dellve

**Affiliations:** ^1^Division of Ergonomics, Department of Biomedical Engineering and Health Systems, School of Engineering Sciences in Chemistry, Biotechnology and Health, KTH Royal Institute of Technology, Stockholm, Sweden; ^2^Department of Sociology and Work Science, Gothenburg University, Gothenburg, Sweden

**Keywords:** workplace health promotion, leadership, interventions, system approach, organizational learning climate

## Abstract

There is limited previous research on how learning processes contribute to the outcomes of workplace health promotion (WHP) leadership interventions. The aim of this study was to identify the outcomes of a system-based WPH education program for managers and investigated what impact the intervention program had on health-oriented leadership, improvement work, and employee well-being, as well as what factors (i.e., how manager's active work following the intervention and organizational learning climate) contributed to these outcomes. A mixed-methods approach was applied, including qualitative interviews with 23 managers and process leaders, as well as questionnaires to employees and managers representing 17 public health care units in Sweden. The results showed that health-oriented leadership, improvement work, work satisfaction, and vitality increased at workplaces that worked actively to implement WHP following the program. Working actively with WHP and health-oriented leadership was of central importance for success and was a covariate with improved social learning climate, improved developmental leadership, and increased degree of improvement work. All included factors of learning during the intervention were associated with improved job satisfaction, while the increase in vitality seemed unrelated to program implementation. In conclusion, successful outcomes of WHP interventions interact with dimensions of organizational learning climate in the workplace.

## Introduction

Despite wide-spread arguments concerning system approaches and leadership involvement for successful workplace health promotion (WHP) interventions (1, 2), there are limited leadership studies about more holistic approaches to employee health (3) and scarce research on how leadership interventions may contribute to improving employee well-being (4). The limited health outcomes of leadership interventions for employees have partly been explained by poor organizational preconditions (5, 6), limited managerial involvement in the planning of intervention content (7, 8), and challenges in capturing and measuring the effects of such holistic approaches (9). This paper sheds light on the organizational learning climate as a proximal process, contributing to the outcomes of a system-based WHP leadership education program. A proximal process is a primary mechanism taking place in an individual's closest context. It includes social interactions that contribute to the development of both the individual and the surrounding environment (10).

### System Approaches to WHP Leadership Interventions

The current knowledge base on how to develop employee health and well-being points at the importance of implementing interventions with a system approach, including broad aspects related to leadership (2, 11). A system-based WHP leadership education program here means a program that integrates how individual, group, organizational, and societal factors are of interrelated importance in developing a workplace setting that promotes health (2, 12, 13). Previous studies have shown that leadership is of particular importance for both handling and affecting such interrelated health factors (14, 15). Developing a leadership program to improve employee health and well-being requires the strengthening both of leaders' broader awareness of interrelated individual and workplace conditions and leaders' prioritization of employee health. This study will focus on such *health-related leadership* as an outcome of WHP leadership interventions, defined by managers' consideration and prioritization of employee health and well-being (2, 16, 17).

There is extensive evidence to suggest that leadership affects many aspects of employees' health, including ratings of psychological well-being (55), job satisfaction (18), vitality (19), stress (20), depression symptoms (21), or healthy work attendance (14). The present study focused on *job satisfaction* and *vitality* as outcome measures of a system-based WHP leadership education program, representing employees' feelings of well-being. Job satisfaction includes here both being content with one's job and being content with specific aspects related to work such as the work environment, future prospects, and development opportunities (56). Vitality has been defined as the experience of having a high degree of energy in combination with a low degree of exhaustion (56).

Previous studies on leadership development programs have pointed out the various, and sometimes even limited, effects on employee health (22–24). More limited results of leadership interventions may be due to, among other causes, the offering of education programs as separate courses not linked to daily leadership practice (25, 26). Organizational preconditions for health-oriented leadership may also be a reason for limited effects. Specifically, managers within the public sector have reported adverse conditions for developing a health-oriented leadership due to clashes between continuous top-down governed rationalizations and opportunities to support employees to perform work according to professional and ethical standards (5, 8, 27). This suggests that interventions should simultaneously support employee health, engagement, productivity, and efficiency. This study thus concerns how a system-based WHP leadership education program contributes to *the work of improvement* in the workplace, including improvements in the psychosocial work environment, efficiency of work processes, and quality of work.

### WHP Leadership Interventions and Organizational Learning Climate

A longitudinal qualitative study by Gustavsson and Ekberg (28) has shown that analyzing a combination of learning processes and health-promoting processes can facilitate the understanding of changes taking place following WHP programs. There are, however, limited previous empirical studies that have investigated how specific learning processes affect the outcomes of workplace health interventions. (57) have performed a qualitative study of how learning factors in WHP interventions contribute to empowerment. Their study showed the importance of employees' reflections on their own well-being, shared insights into the work situation, and group coherence (57). The study presented in this article focused on how the organizational learning climate contributes to the outcomes of system-based WHP leadership education program. Organizational climate is a climate that enables learning to take place (29). It has been highlighted that successful WHP interventions need to be adapted and tailored to the context of the local workplace (12). A critical factor for leadership development work is supporting managers' active work to transfer the teachings from the program to their organizational context (26, 30). This argues for the broader learning of the managers themselves, but also for their competence to create such learning processes in their local units in practice through developmental-oriented leadership. Developmental leadership has been defined as a leadership style that is supportive and motivates employees' growth and development by providing a work environment that facilitates learning (31, 32). This kind of leadership has also been associated with employee health (33). In this study, *developmental leadership* is thus seen as an important part of the organizational learning climate that contributes to the outcomes of a WHP leadership intervention and is defined by a leadership style that provides and prioritizes good development opportunities for employees (56).

The importance of process evaluation has been argued for clarifying critical factors of successful implementation (12). Previous research has thus focused on the factors that hinder or facilitate the implementation of WHP (34), including, for example, having a participatory approach that includes both first-line managers and employees in the planning of the intervention (35), combining bottom-up engagement from employees with top-down managerial support (36), first-line managers clearly prioritizing and delimiting the implementation of feasible program components (2, 14), and a learning climate that promotes improvements (37). A general social learning climate, in the form of social capital in the workplace, can be important for employee health (38) and also support crucial collaboration and engagement in workplace developments that are important for healthy work conditions (39–41). This study will thus focus on the *social learning climate* as an important aspect of the organizational learning climate that contributes to the outcomes of a WHP leadership intervention. The social learning climate, including social capital, is in this study defined and measured by the trusting and reciprocal relationships, both vertical and horizontal, in the organization, as well as collaboration and mutual responsibilities in developing work for the common good (42).

Besides developmental leadership orientation and learning climate, the importance of clear organizational goals and structures for organizational learning for WHP have been highlighted (43). This includes the importance of management structures that integrate systematic improvements to the work environment with ordinary development work (44), a process that requires regular workplace meetings. Such meetings can promote health by facilitating a continuous dialogue; they can also influence developments in the workplace in parallel with work environment issues (45). This study will thus include both continuous *dialogue* at the workplace about work environment issues (43) and *goal clarity* in the form of employees having a clear understanding of workplace goals and what is expected from them at work (58) as potentially important dimensions of the organizational learning climate that may contribute to the outcomes of a WHP leadership intervention. Managers' goal clarity has previously been shown to contribute to healthy work attendance (14, 46). Otherwise, there is, to our knowledge, no quantitative study that has investigated if and how dialogue in the workplace and goal clarity, as dimensions of organizational climate, contribute to WHP outcomes.

*The aim* of this study was to identify the workplace outcomes of a system-based WPH education program for managers, with a particular focus on how unit managers approached the intervention and how learning processes—in the form of the dimensions of the organizational learning climate—contributed to different outcomes. A previous study of the education program showed that managers perceived positive impacts on their leadership and development work following participation in the program, and they described the program as comprehensive, relevant, and useful (2). This study further investigated what impact the intervention program had on a health-oriented leadership, improvement work, and employee well-being, as well as what learning processes (i.e., how the manager's active work following the intervention and the dimensions of organizational learning climate such as developmental leadership, social learning climate, dialogue, and goal clarity) contributed to the results. Based on the content of the leadership program, the following processes affecting the outcomes are assumed:

- Managers change to a more health-oriented leadership following the intervention, which in turn may affect employee well-being.
- Managers involve employees in developing the work environment following the interventions. These participatory learning approaches and the subsequent improvements may affect employee well-being.
- Learning processes and health-promoting processes are interrelated, both with each other and with the organizational context. Increased learning can thus be an outcome of the intervention, but can also simultaneously be an organizational pre-condition contributing to health-oriented leadership and/or improved employee well-being.

## Methods

### Study Design

This mixed-methods study analyzed the implementation and outcomes from a WHP intervention study for leaders in public health care, during the period 2014–2017, with qualitative interviews of the process leaders and managers (*n* = 23) and follow-up questionnaire data answered by the employees (T1, *n* = 346; T2, *n* = 293; T3, *n* = 208) representing 17 public health care units located in two regions in Sweden. More specifically, the analysis focused on how managers' active work and learning processes in the workplace contributed directly and indirectly to different outcomes following the education program. The effects on employees' perceptions of leadership, the learning environment, improvement work, and health following the interventions were analyzed. Informed consent was applied in all data collection. The study was approved by the Central Ethical Review Board at Karolinska Institutet, Stockholm, Sweden (EPN 2014/1883-31/5).

### The Intervention

The WHP interventions were based on an education program and were applied to three groups of managers during six half-day meetings. Two process leaders from HR and/or Occupational Health and Safety (OHS) introduced themes and triggered topics to facilitate participant dialogue, discussions, and reflections on how to integrate the content of the program into daily managerial practices. The WHP program was developed based on literature reviews (2, 47), own research (2, 14, 48), expert reviews, and in an iterative and participatory process that included input from ~500 managers, organizational key actors, and process leaders in 30 different seminars and workshops before, during, and after the interventions. The core idea of the education program is to integrate evidence-based knowledge on how to improve working conditions and health in daily leadership practice. The program was based on working material on the following themes (2):

- health and work engagement;
- how to build health-promoting working conditions;
- how to decrease and prevent risk factors in work;
- strategies for balance and recovery;
- leadership and management to support well-being and engagement;
- co-working and well-functioning work groups;
- how to lead sustainable development work; and
- structures to improve health, the work environment and sustainable developments.

Each theme in the working material included summaries of important theories and evidence-based research, as well as dialogue questions and exercises aimed to support manager in integrating the content of the theme into their own managerial work practice in their workplace. An overarching focus was the interaction between individual and group and the organizational factors that contribute to improving health and preventing health risks. The working material also guided managers in building organizational capacity and resources for dealing with demands within the organization. This included support for managers' action plans to develop general good working conditions, a learning climate, systematized health and occupational management; integrating values and norms for health and well-being into management, and communication to support improvement, coordination; and the building of trust across individual and groups at different organizational levels. The theoretical foundations of and pedagogical ideas for the program are described in greater detail in Dellve and Eriksson (2).

### Data collection and sample

Data were collected via qualitative interviews and questionnaires; 23 managers and process leaders were interviewed in 12 individual interviews and four focus groups during and just after the interventions. The average length of the individual and the focus group interviews were 60 min. The interviews focused on the participants' perspectives on the content of the program, the parts of the program the managers had implemented, and the factors hindering and facilitating implementation. All interviews were recorded and transcribed verbatim.

Staff questionnaires were distributed to all subordinates of the participating managers at baseline (before the intervention, T1), the first follow-up (5 months post intervention period, T2), and the second follow-up (9 months post intervention period, T3). All subordinates employed at T1, T2, respectively, T3 were invited to participate. Each participant was given a unique code to enable to follow the answers of the same respondent over time. Fifteen of the 17 workplaces participated in the intervention study until the last follow up. The response rate was 62–72% (T1, *n* = 346; T2, *n* = 293, and T3, *n* = 208). At T1, 88% of the respondents were female; 38% were registered nurses, 28% were assistant nurses, and 13% were dental nurses. Other professional groups (<10%) included administrators, dentists, and speech therapists. The managers' questionnaire was distributed at the same time points as the staff questionnaire to managers of the 17 units (response rate: 86–100%). For this study a selection of variables from the staff questionnaire was chosen for measuring intervention outcomes and different dimensions of organizational learning climate (see selected variables listed below). All variables selected for this study were measured T1, T2, and T3. The full questionnaire can be distributed by request to the authors.

### Variables

The following *outcome* variables in the staff questionnaire were analyzed:

- *Health-oriented leadership*: two items from a leadership quality instrument (56), including the experience that the leader (1) cares about staff and considers individual needs, (2) prioritizes positive general workplace conditions, and one item including the experience that the leader (3) highly prioritizes employees' health (Cronbach's alpha = 0.91)
- *Improvement work* (49): three items on whether the psychosocial work environment, efficiency of work performed at the work unit, and quality in work performed at the work unit improved in the last 6 months (Cronbach's alpha = 0.89)
- *Vitality, four* items [Cronbach's alpha 0.88; from Copenhagen Psychosocial Questionnaire, (56)]
- *Job satisfaction*, 6 items [Cronbach's alpha = 0.84; from Copenhagen Psychosocial Questionnaire, (56)].

The following variables of *organizational learning climate* in the staff questionnaire were analyzed:

- *Developmental leadership*: two items from the leadership quality index [Copenhagen Psychosocial Questionnaire, (56)]
- *Social learning climate*: five items on the presence of a trusting and collaborative work environment for innovation and one item on trust in higher management [from index on Social capital (42); Cronbach's alpha = 0.89]
- *Goal clarity*: three items from an index on sustainable employee engagement [(58) Cronbach's alpha = 0.76]
- *Dialogue*: three items on continuous dialogue at the workplace on the psychosocial work environment, the physical work environment and planning and development of work (Cronbach's alpha = 0.87).

The job satisfaction scale had a 4-point response scale (1 = *very dissatisfied*, 4 = *very satisfied*). All other items had a 5-point response scale (1 = *to a very low degree*, 5 = *to a very high degree*). The response scales for health-oriented leadership, developmental leadership, goal clarity, and vitality were transformed to a range of 0–100, where 100 represented a very high degree. The results of the staff questionnaire in each workplace (i.e., mean values) were reported to the participating managers directly after each time point measurement. Managers were, with support from the researchers and process leaders, included in the results from the questionnaires when developing intervention action plans. All variables were normally distributed. Histograms and normal quantile plots were used to check whether the variables were normally distributed.

From the managers' questionnaire, only their ratings on the intervention's impact on leadership execution and activity in development work (2 items), as well as an open-ended question on how the interventions had affected their leadership and development work were analyzed.

### Analysis

The generated data was analyzed stepwise. First, a Student's *t*-tests of differences between mean values at T1 (baseline) and T2 as well as between T1 and T3 was used to determine if there were any statistically significant differences in all included measures between T1 and T2 and T1 and T3. Second, the data from the qualitative interviews and the manager questionnaire were used to analyze what actions participating managers implemented following the interventions, as well as the managers' qualitative descriptions of factors affecting implementation. After the qualitative analyses, the participating workplaces were categorized according to whether or not (1/0) they had managers who had been working actively according to the intervention (30). Following these categorizations, Student's *t*-tests of differences in mean values for all included measures at T1, T2, and T3 between these two categories of workplaces were performed. Finally, to analyze how learning processes contributed to outcomes following the intervention, five linear regression models were performed. Independent variables in all five models were differences between T1 and T3 in the ratings for health-oriented leadership, improvement work, job satisfaction, and vitality. The outcomes were stepwise regressed on the dependent variables working actively (1/0) and the differences between T1 and T3 in the ratings for dialogue, goal clarity, social learning climate, and developmental leadership. This meant that linear regression models included: (1) working actively, (2) working actively and differences in dialogue, (3) working actively and differences in dialogue and goal clarity, (4) working actively and differences in dialogue, goal clarity and social learning climate and, (5) working actively and differences in dialogue goal clarity, social learning climate and developmental leadership.

Statistical significance was considered when *p* < 0.05.

## Results

Changes in outcomes and learning factors following the intervention.

Vitality, dialogue, and goal clarity increased over time following the interventions (see Table 1). Health-oriented leadership decreased at the first follow-up, but no statistically significant changes in leadership ratings could be seen over time (see Table 1).

**Table 1:** Mean values at baseline (T1) and the difference (diff) in how individuals rated the factors between T1–T2 and T1–T3.

	**Mean T1 (SE)**	**Diff T1-T2 (SE)**	**Diff T1-T3 (SE)**
Health-oriented leadership	62.1 (24.1)	−4.2** (16.9)	−0.6 (19.2)
Improvement work	3.3 (0.8)	0.0 (0.8)	0.1 (0.9)
Vitality	58.8 (19.8)	4.2** (15.5)	3.7 (17.5)**
Job satisfaction	64.6 (15.9)	−0.1 (12.7)	0.1 (12.2)
Dialogue	3.3 (0.9)	0.1 (0.9)	0.2 (0.9)**
Goal clarity	74.6 (19.2)	3.2 (17.4)**	4.0 (17.4)**
Social learning climate	3.8 (0.7)	0.0 (0.6)	0.0 (0.7)
Developmental leadership	61.3 (23.5)	−0.7 (19.5)	0.4 (20.7)

*SE, standard errors*.

** *p < 0.01*.

### Managers Working Actively Following the Interventions

A majority of participating managers stated that they had become more conscious of their own leadership practices following the intervention program. The program was described as giving insight into the importance of focusing on positive resources at the workplace and providing knowledge about structured and holistic approaches to improve the work environment. The program content was acknowledged as relevant for all managers interviewed, and a majority stated that the program gave them an awareness of the importance of leadership, as well as inspiration for new approaches for handling work environment issues.

*Through reflections and discussions* [during the program], *I have become more aware of my leadership style and how it can have consequences for employee health* (Answer to open-ended question in managers' questionnaire).

The interviews revealed that concrete actions in the workplace following the interventions were rather limited for most participants. Managers noted that their ongoing efforts to solve challenging work conditions hindered them from prioritizing working according to the interventions. Hindrances in the working conditions included time-consuming re-organizations, ongoing workplace conflicts among subordinates, understaffing, or problems in recruiting competent personnel. Some of the participating managers also struggled with their own working conditions as a manager, and some managers became sick, burnt out, or decided to quit as a manager during the intervention period.

*The education program has ended now and I can say that we haven't yet started to do anything following the program. […] It [WHP] has somehow been down prioritized; it [the prioritization of work] has more been about surviving and solving the most urgent problems*. (Unit manager, focus group interview).

A few of the participating managers (*n* = 5, altogether responsible for 108 employees) noted in the interviews that they had been working more actively (i.e., to a greater extent than the others) according to the intervention program. These managers focused on concrete developments in their own leadership style, engaging employees in work environment developments, and developing better structures for work environment improvements, which included engaging subordinates in planning, structuring, and visualizing the needed systematic work environment of the unit (e.g., when discussing the results from the employee survey at staff meetings and based on the discussions decided on what actions to take). Other examples of activities that the managers implemented included staff activities to create a better atmosphere or follow-up on work-life balance among employees.

*I have become more observant of the importance of well-being, that my presence makes a big difference, [I have become] better at seeing the needs of the staff* . (Answer to open-ended question, manager questionnaire).

*I have started to work in a more structured way with workplace health promotion [following the education]*. (Unit manager, focus group interview).

### Changes in Outcomes and Learning Factors Following Active Work

Workplaces working actively had, at baseline, higher ratings for health-oriented leadership (*p* < 0.001), developmental leadership (*p* = 0.04), improvement work (*p* = 0.01), and social learning climate (*p* = 0.02). Over time, the ratings for health-oriented leadership, improvement work, work satisfaction vitality, dialogue, and goal clarity increased at the workplaces working actively (see Table 2). At workplaces not working actively according to the intervention program only vitality increased at the first follow-up and ratings of health-oriented leadership decreased over time (see Table 2).

**Table 2:** Separate analysis of workplaces working actively/not working actively with WHP.

	**Working Actively with WHP**			**Not Working Actively with WHP**		
	**Mean T1 (SE)**	**Diff T 1-T2 (SE)**	**diff T 1-T3 (SE)**	**Mean T1 (SE)**	**Diff T 1–T2 (SE)**	**Diff T 1–T3 (SE)**
Health-oriented leadership	65.0 (23.2)	−0.1 (16.9)	6.3** (15.9)	60.9 (24.4)	−6.4** (16.6)	−4.5* (19.9)
Improvement work	3.4 (0.9)	0.1 (0.74)	0.4** (0.8)	3.2 (0.8)	<0.1 (0.8)	0.1 (0.6)
Vitality	58.7 (20.2)	6.3** (16.1)	6.4** (16.4)	58.8 (19.7)	3.2** (15.1)	2.3 (17.9)
Job satisfaction	65.2 (16.1)	0.2 (12.7)	3.9* (12.5)	64.4 (15.8)	−0.3 (12.8)	−2.1 (11.5)
Dialogue	3.5 (0.9)	0.2 (0.9)	0.4** (0.72)	3.3 (0.9)	<0.1 (0.9)	<0.1 (0.8)
Goal clarity	74.6 (21.1)	4.69* (18.6)	6.5** (16.6)	74.5 (18.4)	2.5 (16.8)	2.6 (17.5)
Social learning climate	3.9 (0.7)	0.1 (0.6)	0.1 (0.5)	3.8 (0.7)	−0.1 (0.6)	−0.1 (0.6)
Developmental leadership	63.6 (23.3)	1.0 (20.2)	4.2 (17.7)	60.4 (23.6)	−1.5 (19.1)	−1.9 (22.1)

*Mean values at baseline (T1) and the difference (diff) in how individuals rated the factors between T1–T2 and T1–T3*.

*SE, standard errors*.

*p < 0.05 and **p < 0.01.

### Learning Factors Contributing to the Outcomes

Table 3 presents outcomes regressed on working actively, dialogue, goal clarity, social learning climate, and developmental leadership stepwise in model 1–5. Model 1 shows that working actively impacted all outcomes, expect vitality. All explaining factors, except dialogue were associated with an increased degree of improvement work in the linear regression models 1–4 (Table 3). However, in the final model (model 5), working actively and improved developmental leadership were the factors that remained associated with an increased degree of improvement work. All factors, except for goal clarity in model 4 and 5, were associated with improved health-oriented leadership in the different models. Except for improved dialogue in models 4 and 5, all factors were also associated with improved job satisfaction. None of the included factors were of statistically significant importance for improved vitality following the intervention.

**Table 3:** The associations of working actively, dialogue, goal clarity, social learning climate, and developmental leadership with the outcomes improvement work, health-oriented leadership, job satisfaction and vitality: The results of multivariate linear regression analysis.

	**Improvement work^†^ β (SE)**	**Health-oriented leadership^†^ β (SE)**	**Job satisfaction^†^ β (SE)**	**Vitality^†^ β (SE)**
**Model 1**				
Working actively	0.2** (0.1)	5.4** (1.5)	3.0**(0.97)	2.0 (1.4)
Intercept	0.2	0.9	−0.9	4.3
Adj *r*^2^	0.1	0.1	0.1	<0.1
**Model 2**				
Working actively	0.2** (0.1)	4.7** (1.5)	2.3** (1.0)	1.3 (1.4)
Dialogue^†^	0.1 (0.1)	6.1** (1.7)	3.3* (1.1)	2.9 (1.6)
Intercept	0.1	−0.6	0.3	3.9
Adj *r*^2^	0.1	0.1	0.1	<0.1
**Model 3**				
Working actively	0.2** (0.1)	3.95** (1.49)	1.95*(0.99)	0.80 (1.43)
Dialogue^†^	0.1 (0.1)	4.90** (1.71)	2.40* (1.09)	1.71 (1.65)
Goal clarity^†^	<0.1* (<0.1)	0.3** (0.1)	0.3** (0.1)	0.2^‡^ (0.01)
Intercept	0.1	−1.7	−0.4	2.9
Adj *r*^2^	0.1	0.2	0.2	0.1
**Model 4**				
Working actively	0.2** (0.1)	3.19* (1.45)	1.94* (0.97)	0.57 (1.43)
Dialogue^†^	0.1 (0.1)	4.32** (1.63)	1.90 (1.08)	1.89 (1.44)
Goal clarity^†^	<0.1 (<0.1)	0.1 (0.1)	0.1** (<0.1)	0.2^‡^ (0.1)
Social learning climate^†^	0.1 (0.1)	12.1** (2.5)	5.5** (1.6)	0.5 (2.5)
Intercept	0.1	−1.0	−0.1	3.2
Adj *r*^2^	0.1	0.2	0.3	<0.1
**Model 5**				
Working actively	0.2** (0.1)	3.0* (1.23)	1.8* (0.9)	0.7 (1.5)
Dialogue^†^	0.1 (0.1)	3.1* (1.4)	1.6 (1.1)	1.9 (1.6)
Goal clarity^†^	<0.1 (<0.1)	<0.1 (0.1)	0.1* (<0.1)	0.1 (0.1)
Social learning climate^†^	0.1 (0.1)	8.5** (2.3)	4.3** (1.6)	0,0 (2.6)
Developmental leadership^†^	0.01* (<0.1)	0.4** (0.1)	0.1** (<0.1)	0.1 (0.1)
Intercept	0.1	−0.4	0.1	3.3
Adj *r*^2^	0.2	0.5	0.3	0.0

*β (SE)= Unstandardized b-coefficients (standard errors)*.

† *Increased/improved health-oriented leadership, improvement work, job satisfaction, vitality dialogue, goal clarity, social learning climate, or developmental leadership = Differences in ratings between T1 and T3*.

‡ p < 0.10, ^*^p < 0.05, and **p < 0.01.

## Discussion

It has been suggested that a combination of analyzing learning processes and health-promoting processes can facilitate the understanding of why changes take place following of WHP programs (28). This study analyzed interrelated outcomes of a system-based WHP leadership education program, including the importance of the manager's active work and the organizational learning climate. The studied education program had a comprehensive approach to WHP and supported the managers in developing action plans for the most important work environment issues in their workplaces. The results showed that health-oriented leadership, improvement work, work satisfaction and vitality increased at workplaces that worked actively to implement WHP following the program. The overall results may demonstrate that the system-based WHP program supported managers to integrate issues of well-being and health into their routine leadership practices. The results thus suggest that comprehensive education programs can facilitate for workplaces to select measures reflecting their relevant needs and local context (2).

Working actively with WHP improved the social learning climate and developmental leadership, which contributed to increased improvement work in the workplace. The WHP activities implemented by the managers differed between each workplace and were based on the managers' judgments on the specific actions relevant to their workplace. These findings further support previous findings that that successful WHP interventions need to be adapted and tailored to the pre-conditions and needs of the local workplace (12). The employees' perceptions of an improved social learning climate and developmental leadership probably resulted from their managers engaging employees in improvement work at the workplace and that these processes contributed to an increase in the learning climate. Of the learning factors, developmental leadership was of specific importance for increased improvement work. This is in line with earlier work that developmental leadership can provide a work environment that supports and motivates learning (31, 32), which can be seen as a pre-condition for improvements in the work environment. It is noteworthy that the different factors included in the analysis only explained 15% of the variance in increased improvements, which means that other factors (not included in this study of WHP) are essential for an increased degree of improvement work. Improvement work in Swedish healthcare is often mandated using a top-down approach (50). This means that demands for improvement from higher levels of management also might have contributed to increased improvement work at the workplaces.

The results showed that a more limited number of workplaces worked actively following the program, which the participating managers explained by challenging existing work conditions that hindered them from prioritizing the implementation of the WHP. Limited outcomes of leadership interventions shown in previous research have also been explained by poor or non-conducive organizational pre-conditions (5, 6). This raises concerns about what kind of learning processes may facilitate the implementation of WHP in workplaces where managers are experiencing challenging conditions. The results suggest the importance of managers having the right pre-conditions to implement WHP, including having time for the development of the work. Previous research has pointed out that a trust-based management culture, that is, giving first-line managers the mandate to take decisions over how to organize work, also supports the managers' increased engagement in work environment improvements (51). A need for a management climate where the organization, including, for example, the top management and key actors from human resources, understand the managers' and employees' motivations for learning and needs for support can thus be seen as an important pre-condition for promoting more active work (8). More research is needed, however, on how the work organization, including the top management, can develop a supportive environment for bottom-up initiated improvement work (51). The results also point to the need for intervention programs within healthcare being flexible enough to support managers with more limited pre-conditions by, for example, supporting managers to limit their work and act on the (for them) most urgent issues.

All factors expect goal clarity were associated with improved health-oriented leadership. The results indicate that organizational goal clarity may be in conflict with the employees' own health. This also supports the idea that the development of health-oriented leadership interplays with the wider organizational learning climate, including social and participatory processes such as continuous dialogue, on work environment issues and the social learning climate at the workplace. The results indicate the importance of giving priority to employee health and a collaborative and supportive work climate (42), as well as setting aside time for learning and meaningful development work (43). A good organizational climate and effective communication processes may not only improve employee influence over their health, but also development work, and the workplace meetings may thus have health-promoting value (45). It has been pointed out that there are challenges in capturing and measuring the effects of holistic intervention programs (9). Due to the challenges of evaluating WHP, the importance of process evaluation has been highlighted (12, 34). There are few previous quantitative studies that have identified indicators for process evaluation of WHP (52), but dimensions of the organizational learning climate can be used as important process indicators based on the results from the present study. The policy implications from this study are thus that aspects of participatory processes, including continuous dialogue as well as the social learning climate, are important key indicators for WHP.

All included factors were associated with improved job satisfaction, but the results also showed that an increase in vitality was not a direct result of managers' active WHP work following the education program, nor was it associated with changes in the organizational learning climate. The results thus indicate that the studied WHP leadership education program contributed to an increase in job satisfaction, but that the increase in vitality was unrelated to program implementation. These results might be due to the measurements of job satisfaction being closely linked to actual work conditions and leadership practices, while feelings of high degree of energy/vitality (56) may also be affected by a number of factors external to the workplace, such as seasonal weather changes, private life circumstances, and life-course factors. The policy implications from this study are thus, that job satisfaction is a better outcome measurement of WHP, compared to vitality, for example.

The conditions of the learning climate can be viewed as an important proximal process, as it affected all intervention outcomes (i.e., health-oriented leadership, improvement work and job satisfaction). Still, learning and health-promoting processes (as well as organizational context) were interrelated. Thus, we cannot draw any conclusions on the causal relationships based on the study data. However, the results can be interpreted as showing that the organizational learning climate could be a pre-condition for succeeding with WHP, while other dimensions of the learning climate are outcomes of the WHP program. Workplaces that were working more actively with WHP had, at baseline, higher ratings for developmental leadership and social learning climate, which indicates the importance of the learning climate as a pre-condition for succeeding with WHP. The dialogue on work environment issues and goal clarity increased when the workplaces were working actively, which suggests that these factors are probable outcomes of the managers' active work with WHP.

There are some obvious limitations and strengths with this study. One limitation is that no control workplaces were included to compare outcome patterns. One particular strength is that the study was based on a mixed methods approach, including managers' own descriptions of how they were affected by and took actions following the program. Performing additional interviews with employees could, however, have given even more comprehensive information on the extent to which changes in the organizational climate were a consequence of the intervention program. Observations of workgroup meetings, for example, could moreover, have given more objective information of what, at workplace level, it actually meant to “have a dialogue” and “work actively.”

Another strength was that long-term outcomes were studied by two follow-up measurements, although the decreased number of employees participating in the follow-up questionnaires due to workplace drop outs and staff turnover is another weakness. Intervention studies are time consuming and may be hard to prioritize for health care workplaces with high workloads, which has resulted in the rather limited number of workplaces that worked actively following the education program. Alternatives to extensive staff questionnaires could be single item questions distributed by short message services (SMS) (53) or focus group assessments (54) which in this context could be considered less time consuming for workplaces to participate in intervention studies.

## Conclusions

Conclusions that can be drawn from this study include the fact that the outcomes of WHP leadership interventions interact with dimensions of the organizational learning climate in the workplace. These interactions can be seen as proximal processes that highly depend on individuals' active behaviors, including social interactions between managers and employees in the workplace (10). This study confirms the value of clustered analysis based on manager's active work to trace outcomes following leadership interventions (30). Practical implications from the study include confirmation that dialogue on work environment issues, developmental leadership, and social learning climate may be used as process indicators for development of comprehensive WHP interventions.

## Data Availability Statement

The raw data supporting the conclusions of this article will be made available by the authors, without undue reservation.

## Ethics Statement

The studies involving human participants were reviewed and approved by Central Ethical Review Board at Karolinska Institutet, Stockholm, Sweden, EPN 2014/1883-31/5. The patients/participants provided their written informed consent to participate in this study. Written informed consent was obtained from the individual(s) for the publication of any potentially identifiable images or data included in this article.

## Author Contributions

AE and LD: conception or design of the work, data analysis and interpretation, critical revision of the article, and final approval of the manuscript version being submitted. AE: data collection and drafting the article. All authors contributed to the article and approved the submitted version.

## Conflict of Interest

The authors declare that the research was conducted in the absence of any commercial or financial relationships that could be construed as a potential conflict of interest.
